# Dietary Determinants of Polyunsaturated Fatty Acid (PUFA) Status in a High Fish-Eating Cohort during Pregnancy

**DOI:** 10.3390/nu10070927

**Published:** 2018-07-20

**Authors:** Marie C. Conway, Maria S. Mulhern, Emeir M. McSorley, Edwin van Wijngaarden, J. J. Strain, Gary J. Myers, Philip W. Davidson, Conrad F. Shamlaye, Alison J. Yeates

**Affiliations:** 1 Nutrition Innovation Centre for Food and Health, Ulster University, Coleraine, County Londonderry BT52 1SA, UK; Conway-m7@ulster.ac.uk (M.C.C.); m.mulhern@ulster.ac.uk (M.S.M.); em.mcsorley@ulster.ac.uk (E.M.M.); jj.strain@ulster.ac.uk (J.J.S.); 2 School of Medicine and Dentistry, University of Rochester, 601 Elmwood Avenue, Rochester, NY 14642, USA; Edwin_van_Wijngaarden@URMC.Rochester.edu (E.v.W.); Gary_Myers@URMC.Rochester.edu (G.J.M.); Phil_Davidson@URMC.Rochester.edu (P.W.D.); 3 The Ministry of Health, Mahé, Republic of Seychelles; shamlaye@gmail.com

**Keywords:** pregnancy, dietary patterns, polyunsaturated fatty acids, PUFA, fish

## Abstract

Polyunsaturated fatty acids (PUFA) are essential for neurodevelopment and the developing foetus depends on an optimal maternal status. Fish is a rich source of PUFA. The current study investigated dietary patterns, and associations with PUFA status in a high-fish consuming cohort of pregnant women in the Seychelles. At 28 weeks’ gestation, pregnant women provided a blood sample, from which serum total PUFA concentrations were measured, A Food Frequency Questionnaire (FFQ) and Fish Use Questionnaire (FUQ) were also completed. Principal component analysis (PCA) of dietary information identified four patterns. Regression analyses found dietary pattern 2, containing foods traditionally eaten in the Seychelles e.g., fish, fruit and vegetables was positively associated with serum docosahexaenoic acid (DHA) (β = 0.134; CI = 0.001, 0.022), and serum total *n*-3 PUFA (β = 0.139; CI = 0.001, 0.023) concentrations. Dietary pattern 1, high in processed foods, snacks, white meat and eggs, was not significantly associated with any of the serum PUFA concentrations. The FUQ indicated that fatty fish was associated with EPA status (β = 0.180; CI = 0.001, 0.005) in high consumers. The second dietary pattern, consisting of higher consumption of fish and fruit, was positively associated with *n*-3 PUFA status during pregnancy.

## 1. Introduction

Optimal nutrition is important for pregnant women as the developing foetus relies on maternal intake of nutrients critical for foetal growth and development [1,2]. The *n*-3 long chain PUFA (LCPUFA) docosahexaenoic acid (DHA) and the *n*-6 LCPUFA, arachidonic acid (AA), are required for growth and development of the brain [3,4,5]. These LCPUFA are preferentially transferred from the mother to the developing foetus [6]. DHA is found in high concentrations in the brain; however, the human body manufactures less than 10% of the requirement [7]. AA is not so prominent in the brain but is important for growth [6]. The *n*-3 and *n*-6 precursors both compete for the enzymes that metabolise them to AA or DHA [8]. Evolutionarily, the ratio of *n*-6 to *n*-3 PUFA was 1:1; however, the modern diet has a ratio of approximately 15:1 [8]. Consequently, the majority of DHA must be obtained preformed and fish is the primary source, as most other foods regarded as containing *n*-3 PUFA, (e.g., walnuts and flaxseed), contain the *n*-3 precursor α-linolenic acid (ALA). 

LCPUFA requirements are highest during the third trimester, a point in pregnancy when there are increased needs of the foetus [9]. Higher maternal *n*-3 PUFA status during pregnancy has been associated with lower systolic blood pressure in offspring [10], increased child height [11], and better language development [12,13]. However, *n*-3 PUFA supplementation studies in pregnant women have not consistently supported improved child neurodevelopmental outcomes [14]. Given the possible influence of maternal PUFA status on child outcomes, it is important that the maternal diet has an optimal balance of PUFA intake during pregnancy to maximise benefits in children.

Although there is no definitive recommended amount of total LCPUFA or their individual components to obtain daily during pregnancy, the European Food Safety Authority (EFSA) determined that 250 mg DHA plus EPA, as well as an additional 100 to 200 mg preformed DHA should be consumed [15]. Fish is a rich source of *n*-3 LCPUFA and its consumption is regarded as a major determinant of *n*-3 PUFA status in the body [16,17,18]. The association between fish consumption and PUFA status has been investigated previously in both pregnant and non-pregnant cohorts. Several studies have indicated increased fish consumption was associated with higher *n*-3 PUFA [16,19,20,21,22,23], however, others have found no association [24,25]. The lack of association between fish consumption and PUFA status during pregnancy might be owing to the increased biological transfer of PUFA from mother to foetus during the third trimester.

It is likely that not only fish intake but also other dietary components influence biomarker status. Dietary patterns reflect combinations of foods eaten together. Previous research has demonstrated that dietary patterns are associated with biomarkers for vitamin B12, folate [26], and PUFA [27]. The use of dietary pattern analysis allows dietary habits to be identified, giving an insight into the entire diet, rather than only single foods [2,28,29,30]. Recent studies suggest that foods not considered to be a source of PUFA, such as vegetables, fruits and fruit juice, dairy products and tea, were associated with PUFA status, highlighting that the food matrix effect may influence PUFA status [27,31]. Although this could be a chance finding, this research suggests that PUFA status may be influenced by interactions between constituents of these food items. The aim of the current study is to investigate associations between dietary patterns and maternal PUFA status in a high fish eating cohort of pregnant women.

## 2. Materials and Methods 

### 2.1. Study Characteristics

The Seychelles Child Development Study (SCDS) is an ongoing longitudinal observational study in the Republic of Seychelles, an archipelago of islands in the Indian Ocean. The overall aim of the SCDS is to investigate the influence of prenatal methyl mercury (MeHg) exposure from maternal fish consumption during pregnancy on child development outcomes in a high fish eating cohort [13,32]. Recruitment for Nutrition Cohort 2 (NC2) took place between January 2008 and 2011 on Mahé, the main island of Seychelles. A total of 1535 pregnant women were recruited during their first antenatal visit (from 14 weeks’ gestation) at 8 health centres across the island. 

### 2.2. Dietary Assessment

At approximately 28 weeks’ gestation a food frequency questionnaire (FFQ) was completed to assess dietary intakes, with participants asked to specify the average frequency of consumption for each food item over the previous 6 months (Supplementary file 1). The FFQ was specifically designed for the Seychellois population and was available in English as well as the native Kreol. It included 137 questions on a wide range of food items including local foods and foods commonly eaten in western areas. A subset of participants (*n* = 401) recruited to NC2 completed this FFQ. Frequencies listed on the FFQ ranged from selecting 4–5 times per day, to never consuming the food item. Where appropriate, standard portion sizes were used to describe serving sizes included on the questionnaire, for example, 1 slice of pizza, 1 cup, 1 mug. Completed FFQs were entered on QBuilder software (Tinuviel, version 4.0, Anglesey, UK), which had an updated food and nutrient database which included Seychellois specific foods, allowing for the food and nutrient intake to be analysed. The output from QBuilder converted the frequency scores for each food item to a weekly intake. From this output, g/day was manually calculated by multiplying the frequency score by an average portion size and dividing by 7. Participants also completed a retrospective fish use questionnaire (FUQ) (Supplementary file 2) at 28 weeks’ gestation where they indicated the number of times per week during pregnancy they consumed specific types of fish. The FFQ allows for the estimation of frequency and portion size of food items consumed, including fish. The FUQ supplements this by estimating the number of times per week a variety of fish are eaten. 

### 2.3. Anthropometry

The mothers’ weight and height measurements were recorded by trained nurses when their children were approximately 20 months old. From these anthropometric measurements, body mass index (BMI) (kg/m^2^) was calculated. In a previous cohort (Nutrition Cohort 1) maternal BMI at 20 months was highly correlated (*r* = 0.9) with preconception BMI. To ensure accuracy of measurements all equipment was calibrated by the Seychelles Bureau of Standards prior to the study starting and throughout the duration of the study. 

### 2.4. Under/Over Reporting of Energy Intake 

Energy intake (MJ/d) for each participant was calculated using FFQ data. The accuracy of the estimated energy intake for each participant was assessed to determine the number of under reporters, over reporters, and plausible reporters. Basal metabolic rate (BMR) (MJ/day) was calculated using the Henry equations [33]. A total of 19 participants had missing weight and height values. The mean weights and heights of the study population were used for those participants in order for their BMR to be determined, and their energy intake subsequently assessed for plausible reporting or misreporting. There were no significant differences in participant characteristics between the full cohort (with mean imputation for missing values, *n* = 401 and the subcohort for which no data were missing (*n* = 382) when comparing food group intakes, nutrient intakes and PUFA status (data not shown). The validity of participants reported energy intake, as calculated from the FFQ, was assessed using Goldberg et al. [34] cut offs as outlined by Black [35]. A Physical Activity Level (PAL) of 1.4 was used as recommended by Prentice et al. [36] for pregnant women. Using the calculated cut-offs, participants were labelled as under, over or plausible reporters. 

### 2.5. Dietary Pattern Analysis: Principal Component Analysis 

A total of 18 food groups and their daily consumption (g/day) were manually created. Foods were grouped according to the types of food or by similarities in nutrient content (see Table 1 for food items included in each food group) [28]. Principal component analysis (PCA) with varimax rotation was performed on the 18 food groups. This procedure allowed for the identification of the principal directions in which the data varied, and thus similarities and differences between food groups. The criterion for retaining factors included those with an eigenvalue greater than 1, and on the basis of where the scree plot showed a break or “elbow” (Supplementary Figure S1). Varimax rotation was used to rotate factors, redistributing the explained variance for the individual components and thus enabling interpretation. Dietary pattern scores were then calculated by multiplying the factor loadings generated by PCA by each participant’s food group total and then adding these. These scores were subsequently used as a continuous variable to determine associations with dietary patterns. Food groups with factor loadings greater than 0.3 were considered to significantly contribute to a dietary pattern.

### 2.6. Blood Sampling and PUFA Analysis

At 28 weeks’ gestation, non-fasting maternal blood samples (30 mL) were collected and analysed as previously described [37]. Briefly, blood samples were processed by centrifuging at 2500 rpm for 15 min. Aliquots of serum were stored at −80 °C until analysis. Samples were shipped at −80 °C to Ulster University where serum total PUFA analysis was completed using an adapted method by Folch et al. [38]. PUFA were subsequently detected and quantified by gas chromatography-mass spectrometry (7890A-5975C; Agilent, UK), using Heptadecanoic acid (C17:0) as an internal standard, as described previously [13]. Individual PUFA’s were measured including linoleic acid (LA) (18:2*n*-6), α-linolenic acid (ALA) (18:3*n*-3), AA (20:4*n*-6), EPA (20:5*n*-3) and DHA (22:6*n*-3) and results were presented as mg/mL. Total *n*-6, total *n*-3, and the *n*-6:*n*-3 ratio were also calculated.

### 2.7. Statistical Analysis

Statistical analysis was completed using Statistical Package for Social Sciences (SPSS, version 24, IBM, Chicago, IL, USA). Data for all variables were tested for normality. Normality tests indicated food group, nutrient intake, and PUFA data were not normally distributed. Descriptive analysis was performed and all data expressed as median and interquartile range. Non-parametric statistical tests were completed for comparison of differences between all participants (*n* = 401) and plausible reporters only (*n* = 268). Multiple linear regression analysis was used to examine relationships between dietary patterns and PUFA status while controlling for factors also known to influence PUFA status (maternal age and BMI). Following calculation of energy misreporting, PCA analysis was completed on plausible reporters only (*n* = 268).

Fish intake was further assessed using grams per day (g/day) data from the FFQ (*n* = 268), and also frequency of consumption data from the FUQ (*n* = 260). Participants with FFQ data were ID matched with those with FUQ data. Fish intake was split into tertiles of fish consumption to assess whether low, medium, or high fish intake is associated with PUFA status. Dummy variables were created where tertile 1 (low intake) acted as the reference to which tertile 2 (medium intake) and tertile 3 (high intake) were compared. Regression analysis of fish intake tertile and associations with PUFA status was completed. BMI is known to correlate with PUFA status, with increased BMI being associated with increased *n*-6 PUFA, and decreased *n*-3 PUFA concentrations [39]. Therefore, BMI was considered a potential confounder and was controlled for in our regression analyses. Socioeconomic status was collected using Hollingshead 4-Factor Social Status Index, modified for use in the Seychelles, and was also included as a covariate. Statistically significant results were considered as those with *p*-value < 0.05.

## 3. Results

### 3.1. Subject Characteristics 

A total of 401 participants had complete FFQ and PUFA status data. Characteristics of the participants are presented in Table 2. It was determined that 8 (2.0%) participants were classified as under reporters, 125 (31.2%) as over reporters, and 268 (66.8%) were considered to be plausible reporters. Differences in general characteristics of all participants (*n* = 401) and plausible reporters (*n* = 268) are shown in Table 2. When comparing mean reported intakes of fish for all participants with plausible energy reporters only, intake of “Fatty fish” and “Other fish and fish products” was significantly lower for plausible energy reporters. Nutrient intakes for all participants, and plausible reporters only, as determined from FFQ data, are included in Supplementary Table S1.

### 3.2. Identification of Dietary Patterns 

PCA was completed on plausible reporters only (*n* = 268) because misreporting has previously been suggested to influence study outcomes [40]. Factor loadings for dietary patterns are shown in Table 3. Using the 18 food groups, PCA extracted a total of 4 components with an eigenvalue greater than 1. The scree plot (Supplementary Figure S1) also allowed for the identification of the break point of dietary patterns to be retained. A total of four dietary patterns were identified, explaining in total 36.68% of the variance in the cohort’s diet. The first dietary pattern identified was high in foods considered to be common in a western diet including snacks such as chocolate and crisps, white fish, chicken and eggs and explained 13.45% of the variance. The second dietary pattern accounted for 8.46% of the variance and contained foods traditionally eaten in the Seychelles including fatty fish, other fish and fish dishes, beverages and fruit. The third dietary pattern was characterized by high amounts of crustaceans, molluscs and white fish. This third pattern accounted for 7.61% of the total variance. The fourth dietary pattern explained 7.16% of the total variance and contained red meat, meat products and dishes, and eggs.

### 3.3. Multiple Linear Regression 

Table 4 shows results for multiple regression analysis. Dietary pattern 1, was not significantly associated with PUFA status. The second dietary pattern was significantly positively associated with serum DHA (β = 0.134; CI = 0.001, 0.022), and serum total *n*-3 PUFA (β = 0.139; CI = 0.001, 0.023). The third dietary pattern was not significantly associated with PUFA status. Dietary pattern 4 showed a significant positive association with serum ALA (β = 0.290; CI = 0.001, 0.002), and a significant negative association with serum DHA (β = −0.157; CI = −0.023, −0.003), and total *n*-3 PUFA (β = −0.138; CI = −0.022, −0.002). 

### 3.4. Examining Tertiles of Fish Intake 

Fish intake for fatty, white and total groups based on the FFQ (*n* = 268) and FUQ (*n* = 260) data were categorised by tertiles and regressed against PUFA status using multiple linear regression analyses. For both the FFQ and FUQ data, fish intake was separated into the appropriate fish group. Using the FFQ g/day intake there were no significant associations between tertiles of fish intake and PUFA status (Table 5). Using frequency of fish intake data from the FUQ, fatty fish intake in the high consumers tertile (Tertile 3) was positively associated with EPA status (β = 0.180; CI = 0.001, 0.005) only.

## 4. Discussion

Dietary pattern analysis in this cohort of pregnant Seychellois women identified four main patterns. Dietary pattern 2, described pregnant women with a high intake of fatty fish (e.g., mackerel, tuna, and red snapper), fish dishes (e.g., bouillon blan—a fish soup traditional in the Seychelles), and fruits and beverages. This dietary pattern was significantly associated with serum DHA and total *n*-3 PUFA concentrations. When FUQ data were categorised by tertiles of fish consumption and PUFA, fatty fish intake was associated with EPA status (β = 0.180; CI = 0.001, 0.005) in the highest level of fish consumption (tertile 3). These findings indicate that a diet rich in fish, particularly oily fish, is associated with higher maternal concentrations of *n*-3 LC-PUFA at 28 weeks’ gestation. Dietary intake of *n*-3 PUFA is especially important during pregnancy because of their role in foetal neurodevelopment [41].

Dietary pattern 1 was the most commonly followed pattern in the study population, but did not show any associations with PUFA status. This pattern is high in foods commonly eaten in a “Westernised” diet including processed foods, white meat and eggs. This finding is in line with the transitioning of the Seychelles diet towards a more Westernised diet between 1989 and 2011 [42]. Given that dietary pattern 1 does not appear to be associated with higher PUFA status, there is the potential that *n*-3 PUFA status may be compromised if dietary trends continue to shift in this way. The third dietary pattern did not show any associations with PUFA. The negative association of the fourth dietary pattern, with DHA and total *n*-3 PUFA is similar to that reported previously [43], and is likely owing to a diet high in meat and meat products being associated with higher *n*-6 PUFA than *n*-3 PUFA [44].

Previous research investigating the associations between blood PUFA concentrations and fish consumption has been inconclusive. Some authors report positive associations in non-pregnant [45] and pregnant cohorts [46] while others have found no association [9] between increased fish consumption and PUFA status. Previous research completed as part of the SCDS found that habitual fish intake was not associated with LCPUFA status in pregnant women [25]. Multiple factors may account for the varied results, including cohorts studied and methodological factors. Additionally, pregnancy itself is a complex biological process with preferential transfer of PUFA in the third trimester [25]. Nevertheless, none of these studies analysed for dietary patterns.

Evaluation of dietary patterns allows for the investigation of foods eaten commonly in combinations rather than examining the influence of individual foods [47]. The findings reported here indicate that dietary patterns loading high for fatty fish, fish dishes, fruit and beverages are associated with increased DHA and total *n*-3 status. This dietary pattern approach may be viewed as more representative of how the diet as a whole influences PUFA status. A limited number of such studies during pregnancy are reported. In a cohort of 154 women, Benaim et al. [27] found that pre-pregnancy dietary patterns, as assessed by an FFQ six months prior to gestation, loading high for “healthy” foods were associated with increased *n*-3 fatty acid status during pregnancy. This “healthy” dietary pattern included foods such as vegetables, fruits, fruit juices, fish and dairy products. This finding is consistent with the results of the current study as we concluded a dietary pattern loading high for fish and fruit was associated with increased DHA and total *n*-3 PUFA concentrations. It has been suggested that in a varied diet, foods not regarded to be a rich source of a certain nutrient may increase nutrient bioavailability, with nutrients interacting to increase or decrease bioavailability of one another, alluding to a food matrix effect [27,31]. They found that dietary patterns containing foods that do not have a significant PUFA content such as cabbages, root vegetables, coffee and tea were associated with increased plasma and red blood cell *n*-3 PUFA. This increase in *n*-3 PUFA status may be owing to the phytochemical and antioxidant vitamin content of these foods [31].

It is well established that maternal dietary intake during pregnancy is important for the developing foetus, and subsequent child outcomes [47,48]. Dietary pattern analysis during pregnancy has mainly focused on pregnancy and child outcomes rather than on associations with maternal nutrient status. dos Santos Vaz et al. [49] focused on dietary patterns during pregnancy and anxiety symptoms, while also accounting for *n*-3 fatty acid intake from seafood. They concluded that “health-conscious” and “traditional” dietary patterns showed protective associations for anxiety levels. The influence of maternal dietary patterns on newborn adiposity has also been examined. A dietary pattern with high intakes of potatoes, fats, non-wholegrain foods and vegetables and found it associated with increased newborn adiposity and increased maternal fasting glucose [47]. However, these studies did not analyse blood PUFA concentrations [47,49], and thus could not examine associations between the dietary patterns they identified and PUFA status.

This study has numerous strengths. Dietary assessment included the use of both a FFQ and FUQ to estimate the frequency of consumption of a range of food. The use of these together allowed for a detailed assessment of dietary intakes to be completed. The biological measurement of PUFA rather than solely dietary intake is a further strength. Furthermore, PCA is regarded as the most commonly used method for obtaining dietary patterns. The study also has some limitations. FFQs are reported to be prone to misreporting of intakes, particularly over reporting. The FFQ used here was designed specifically for this study of the Seychellois population, but it has not been validated. The dietary data and blood samples were only collected at one-time point in this study, and may not represent what is happening across the different stages of pregnancy. 

This study’s finding of a dietary pattern consisting of foods traditional to a Seychellois diet being associated with *n*-3 PUFA status is important for public health reasons. The diet in the Seychelles, and likely other developing nations, has been changing over time to become more “Westernised” [42] with a reported decrease in fish consumption [50]. We have previously reported a decline in fish meals per week from 12 meals per week in the Main Cohort [51] to 9 meals per week in NC1 [37] to 8.5 meals per week as reported more recently for the NC2 cohort [13]. Given the importance of *n*-3 PUFA for health, including cardiovascular and immune function and neurodevelopment, following a diet of fish consumption should be optimal for *n*-3 PUFA status.

## 5. Conclusions

Among the four dietary patterns identified, only dietary pattern 2 was associated with DHA and total *n*-3 PUFA status. Women with greater adherence to this dietary pattern had higher fish intake and higher DHA and total *n*-3 PUFA status. The high amounts of fruit and beverages in this pattern may also allude to the potential influence of the food matrix effect on biomarker status. Considering the importance of *n*-3 PUFA to neurodevelopment and other health benefits for the child throughout life such as improved cardiac and immune function, dietary recommendations for fish consumption during pregnancy should consider the importance of fish consumption and its association with increased PUFA status.

## Figures and Tables

**Table 1:** Food groups used for dietary pattern analysis.

Food Group	Food Items in Food Group
Beverages	Fruit juice, water, fizzy drinks, hot drinks (including tea, coffee, hot chocolate, Ovaltine, Milo, Horlicks, Bournvita, Nesquik)
Alcoholic beverages	All alcoholic beverages
Fruit	All fruits, including dried and canned varieties
Vegetables	All vegetables, including potatoes and legumes
Other foods	Other foods, including nuts, herbs and spices, soups, sauces and condiments, and miscellaneous foods such as pizza, biscuits, and cake
Sugars, snacks and preserves	Typical Seychellois snacks: corn snacks, chilli cakes, cassava, as well as other snacks including crisps, chocolate, and sweets
Milk and milk products	All varieties of milk and milk products (yogurt, cheese, milk, ice cream)
Meat products and dishes	Bacon, sausages, frankfurters, burgers, ham, pate, processed meat, black pudding, meat patties, meat organs, pasta with meat, noodles with meat
Fats and oils	Fat and oils used for frying foods
White meat	Chicken
Eggs	Eggs
Red meat	Beef, pork, lamb
Cereals and cereal products	All cereals, including porridge, oatmeal, cornflakes, other breakfast cereals, white bread, brown bread, rice
White fish	All white fish including red snapper, grouper, parrot fish, emperor fish, job, shark, salted fish and spinefoot shoemaker
Fatty fish/oily fish	All fatty fish, including mackerel, tinned tuna, tinned sardines, tuna, karang, bonito, barracuda
Crustaceans	All crustaceans, including prawns, crab and lobster
Other fish and fish products	All other varieties of fish and fish dishes including other fish, bouillon bred with fish, bouillon blan with fish, fish chutney
Molluscs	Octopus

**Table 2:** Characteristics of all participants and plausible reporters only.

	Median (IQR)		
	All Participants *n* = 401	Plausible Reporters Only *n* = 268	*p*-Value
**Age (years)**	26.00 (9.00)	26.00 (8.00)	0.361
**Weight (kg)**	70.80 (23.10)	72.17 (23.28)	0.188
**Height (m)**	1.62 (0.08)	1.62 (0.08)	0.725
**BMI (kg/m^2^)**	27.05 (8.40)	27.49 (7.87)	0.205
**Hollingshead SES**	32.00 (16.50)	33.00 (16.50)	0.187
**Energy intake (kcal/day)**	2763.00 (1794.00)	2326.50 (983.25)	**<0.001**
**Energy intake (MJ/day)**	11.57 (7.51)	9.74 (4.12)	**<0.001**
***Fish intake (g/day)***			
**White fish**	62.57 (84.86)	57.43 (73.71)	0.089
**Fatty fish**	108.86 (117.71)	98.43 (92.57)	**0.027**
**Crustaceans**	1.71 (4.14)	1.71 (4.14)	0.628
**Other fish and fish products**	52.14 (58.93)	43.64 (39.86)	**0.008**
**Molluscs**	5.14 (5.14)	5.14 (5.14)	0.941
***Total serum PUFA (mg/mL)***			
**LA**	0.92 (0.34)	0.93 (0.32)	0.699
**ALA**	0.04 (0.00)	0.04 (0.00)	0.943
**AA**	0.22 (0.10)	0.23 (0.11)	0.639
**EPA**	0.05 (0.00)	0.05 (0.00)	0.809
**DHA**	0.20 (0.11)	0.20 (0.11)	0.959
**Total *n*-6**	1.14 (0.41)	1.14 (0.40)	0.648
**Total *n*-3**	0.29 (0.11)	0.29 (0.12)	0.940
**Mothers *n*-6:*n*-3 ratio**	3.90 (1.21)	3.94 (1.25)	0.698

SES: Socioeconomic status; LA: Linoleic acid; ALA: α-linolenic acid; AA: arachidonic acid; EPA: eicosapentaenoic acid; DHA: docosahexaenoic acid; Differences between groups determined by Mann Whitney U, *p* < 0.05 considered statistically significant.

**Table 3:** Factor loadings for dietary patterns identified by principal component analysis (PCA) for *n* = 268 (plausible reporters only).

Food Group	Dietary Pattern 1	Dietary Pattern 2	Dietary Pattern 3	Dietary Pattern 4
**Other foods**	0.653	-	-	-
**White fish**	0.615	-	0.317	-
**Sugars, snacks, preserves**	0.544	-	-	-
**White meat**	0.516	−0.344	-	-
**Eggs**	0.465	-	-	0.341
**Other fish and fish-products**	-	0.697	-	-
**Fatty fish**	-	0.695	-	-
**Beverages**	-	0.361	-	-
**Crustaceans**	-	-	0.760	-
**Molluscs**	-	-	0.746	-
**Red meat**	-	-	-	0.746
**Meat products and dishes**	-	-	-	0.643
**Vegetables**	-	-	-	-
**Fruit**	-	0.352	-	-
**Cereals and cereal products**	-	-	-	-
**Fats and oils**	-	-	-	-
**Alcoholic beverages**	-	-	-	-
**Milk and milk-products**	-	-	-	-
***% of variance explained***	*13.45*	*8.46*	*7.61*	*7.16*

Only food groups with factor loading values of ≤−0.3 or ≥0.3 were included, some food groups were excluded as they did not load onto any factor retained.

**Table 4:** Multiple regression analysis of dietary patterns as predictors of total serum PUFA status (mg/mL) (*n* = 268) total g/day.

	Dietary Pattern 1		Dietary Pattern 2		Dietary Pattern 3		Dietary Pattern 4	
PUFA (mg/mL)	β	95% CI	β	95% CI	β	95% CI	β	95% CI
**LA**	−0.045	(−0.049, 0.026)	0.089	(−0.011, 0.057)	−0.033	(−0.041, 0.024)	−0.081	(−0.053, 0.011)
**AA**	−0.001	(−0.010, 0.010)	0.067	(−0.004, 0.014)	−0.006	(−0.009, 0.009)	−0.030	(−0.011, 0.007)
**ALA**	−0.054	(−0.001, 0.000)	0.106	(0.000, 0.001)	−0.119	(−0.001, 0.000)	0.290	(0.001, 0.002) *
**EPA**	−0.069	(−0.002, 0.001)	0.045	(−0.001, 0.001)	0.088	(0.000, 0.002)	−0.025	(−0.001, 0.001)
**DHA**	−0.103	(−0.020, 0.003)	0.134	(0.001, 0.022) *	−0.031	(−0.013, 0.007)	−0.157	(−0.023, −0.003) *
**Total *n*-6**	−0.039	(−0.054, 0.030)	0.095	(−0.010, 0.067)	−0.031	(−0.046, 0.027)	−0.078	(−0.060, 0.013)
**Total *n*-3**	−0.108	(−0.021, 0.003)	0.139	(0.001, 0.023) *	−0.028	(−0.013, 0.008)	−0.138	(−0.022, −0.002) *
***n*-6:*n*-3 ratio**	0.058	(−0.144, 0.340)	−0.059	(−0.320, 0.121)	0.018	(−0.179, 0.240)	0.055	(−0.116, 0.301)

Multiple linear regression adjusting for age, BMI, energy intake, and Hollingshead socioeconomic status; * significant results *p* < 0.05.

**Table 5:** Association between serum PUFA status and tertiles (T) of fish intake (grams per day) for all reporters and plausible reporter.

	FFQ g/day Intake (Plausible Reporters *n* = 268)					FUQ Frequency of Fish Intake (Plausible Reporters *n* = 260)				
	T1 (Low)	T2 (Medium)		T3 (High)		T1 (Low)	T2 (Medium)		T3 (High)	
		Beta	95% CI	Beta	95% CI		Beta	95% CI	Beta	95% CI
**White fish**										
**LA**	Ref	−0.117	(−0.132, 0.002)	−0.030	(−0.084, 0.051)	Ref	−0.016	(−0.078, 0.060)	0.030	(−0.054, 0.089)
**AA**	Ref	−0.039	(−0.025, 0.013)	0.009	(−0.017, 0.020)	Ref	0.041	(−0.013, 0.026)	0.032	(−0.015, 0.025)
**ALA**	Ref	0.032	(−0.001, 0.002)	−0.097	(−0.002, 0.000)	Ref	0.016	(−0.001, 0.001)	0.110	(0.000, 0.002)
**EPA**	Ref	0.006	(−0.002, 0.002)	0.033	(−0.001, 0.002)	Ref	0.010	(−0.002, 0.002)	0.032	(−0.001, 0.002)
**DHA**	Ref	−0.027	(−0.026, 0.016)	−0.037	(−0.027, 0.015)	Ref	0.026	(−0.017, 0.026)	0.056	(−0.012, 0.033)
**total *n*-6**	Ref	−0.112	(−0.147, 0.005)	−0.024	(−0.092, 0.061)	Ref	−0.004	(−0.081, 0.075)	0.034	(−0.058, 0.104)
**total *n*-3**	Ref	−0.023	(−0.026, 0.017)	−0.038	(−0.029, 0.015)	Ref	0.026	(−0.017, 0.027)	0.064	(−0.011, 0.035)
**n6:*n*-3 ratio**	Ref	−0.041	(−0.580, 0.288)	0.009	(−0.401, 0.468)	Ref	−0.031	(−0.555, 0.334)	−0.022	(−0.544, 0.381)
**Fatty fish**										
**LA**	Ref	−0.008	(−0.072, 0.063)	−0.021	(−0.079, 0.056)	Ref	0.024	(−0.054, 0.078)	−0.060	(−0.116, 0.041)
**AA**	Ref	0.011	(−0.017, 0.020)	0.001	(−0.019, 0.019)	Ref	0.022	(−0.015, 0.022)	0.024	(−0.018, 0.026)
**ALA**	Ref	−0.027	(−0.001, 0.001)	0.075	(0.000, 0.002)	Ref	−0.091	(−0.002, 0.000)	0.064	(−0.001, 0.002)
**EPA**	Ref	−0.050	(−0.003, 0.001)	−0.012	(−0.002, 0.002)	Ref	−0.113	(−0.003, 0.000)	0.180	(0.001, 0.005) *
**DHA**	Ref	0.010	(−0.019, 0.023)	−0.012	(−0.023, 0.019)	Ref	−0.006	(−0.022, 0.020)	0.018	(−0.021, 0.028)
**total *n*-6**	Ref	−0.005	(−0.079, 0.073)	−0.018	(−0.088, 0.065)	Ref	0.026	(−0.059, 0.090)	−0.046	(−0.122, 0.055)
**total *n*-3**	Ref	0.004	(−0.021, 0.022)	−0.009	(−0.023, 0.020)	Ref	−0.020	(−0.025, 0.018)	0.036	(−0.018, 0.033)
**n6:*n*-3 ratio**	Ref	−0.056	(−0.636, 0.234)	−0.018	(−0.499, 0.374)	Ref	0.002	(−0.421, 0.432)	−0.014	(−0.564, 0.452)
**Total all fish**										
**LA**	Ref	−0.048	(−0.094, 0.041)	−0.038	(−0.088, 0.046)	Ref	0.023	(−0.057, 0.082)	−0.077	(−0.119, 0.028)
**AA**	Ref	−0.010	(−0.021, 0.017)	0.020	(−0.016, 0.022)	Ref	0.019	(−0.016, 0.022)	0.029	(−0.016, 0.026)
**ALA**	Ref	0.001	(−0.001, 0.001)	0.034	(−0.001, 0.002)	Ref	−0.027	(−0.001, 0.001)	0.109	(0.000, 0.002)
**EPA**	Ref	−0.045	(−0.003, 0.001)	0.044	(−0.001, 0.003)	Ref	−0.014	(−0.002, 0.002)	0.095	(0.000, 0.004)
**DHA**	Ref	−0.050	(−0.030, 0.012)	−0.005	(−0.022, 0.020)	Ref	−0.076	(−0.035, 0.008)	0.077	(−0.008, 0.038)
**total *n*-6**	Ref	−0.044	(−0.105, 0.049)	−0.028	(−0.094, 0.058)	Ref	0.024	(−0.063, 0.094)	−0.060	(−0.124, 0.043)
**total *n*-3**	Ref	−0.052	(−0.031, 0.012)	0.001	(−0.022, 0.022)	Ref	−0.076	(−0.036, 0.008)	0.089	(−0.006, 0.041)
**n6:*n*-3 ratio**	Ref	0.031	(−0.325, 0.548)	−0.043	(−0.588, 0.283)	Ref	0.117	(−0.022, 0.867)	−0.121	(−0.933, 0.012)

T1 = tertile 1, T2 = tertile 2, T3 = tertile 3; T1 was reference to which T2 and T3 were compared; regression models adjusted for maternal age at enrolment, maternal BMI, and Hollingshead socioeconomic status; * significant results *p* < 0.05.

## Supplementary Materials

The following are available online at http://www.mdpi.com/2072-6643/10/7/927/s1, Figure S1: Scree plot for identifying dietary patterns from principal component analysis, Table S1: Comparison of daily energy and nutrient intakes with UK DRV for all participants (*n* = 401) and plausible reporters only (*n* = 268), File S1: Seychelles child development study NC2 diet, File S2: Seychelles child development study NC2 fish use questionnaire.
