# Supplementary material for: Dietary Determinants of Polyunsaturated Fatty Acid (PUFA) Status in a High Fish-Eating Cohort during Pregnancy
Source: Nutrients. 2018 Jul 20;10(7):927. doi: 10.3390/nu10070927 (PMC6073891; doi:10.3390/nu10070927)
Supplement: Supplementary file 1 [file nutrients-10-00927-s001.zip › Supplementary Table 1.docx]

**Supplementary Table 1.** Comparison of daily energy and nutrient intakes with UK DRV for all participants (n=401) and plausible reporters only (n=268).

|  | **Median (IQR)** | |  |  |  |
| --- | --- | --- | --- | --- | --- |
|  | **All participants**  **n=401** | **Plausible reporters only n=268** | ***p*-value** | **UK RNI** | **UK EAR** |
| **Energy (kcal)** | 2763.00 (1794.00) | 2326.50 (983.25) | **<0.001** |  |  |
| **Energy (MJ)** | 11.57 (7.51) | 9.74 (4.12) | **<0.001** |  | 9.90 |
| **Protein (g)** | 122.50 (78.90) | 102.95 (47.05) | **<0.001** | 51.00 |  |
| **Protein (% energy)** | 17.37 (4.11) | 17.51 (3.96) | 0.955 |  |  |
| **Fat (g)** | 106.70 (69.20) | 88.70 (44.58) | **<0.001** |  |  |
| **Fat (% energy)** | 35.18 (6.27) | 34.69 (6.47) | 0.404 |  | 35.00(DRV) |
| **Carbohydrates (g)** | 353.40 (236.65) | 298.25 (136.23) | **<0.001** |  |  |
| **CHO (% energy)** | 50.34 (7.70) | 50.71 (7.53) | 0.482 |  | 50.00(DRV) |
| **Saturated fat (g)** | 44.80 (32.35) | 36.55 (19.83) | **<0.001** |  |  |
| **Saturated fat (% energy)** | 14.70 (4.54) | 14.54 (4.32) | 0.262 |  | 10(DRV) |
| **Monounsaturated fat (g)** | 35.20 (22.55) | 29.10 (13.80) | **<0.001** |  |  |
| **Polyunsaturated fat (g)** | 16.80 (11.20) | 14.15 (7.25) | **<0.001** |  |  |
| **Sugar (g)** | 176.30 (141.80) | 141.75 (77.15) | **<0.001** |  |  |
| **Sodium (mg)** | 3106.00 (2081.00) | 2614.00 (1307.25) | **<0.001** | 1600.00 |  |
| **Potassium (mg)** | 3922.00 (2974.00) | 3315.00 (1681.25) | **<0.001** | 3500.00 |  |
| **Calcium (mg)** | 1411.00 (1353.50) | 1139.00 (751.25) | **<0.001** | 700.00 | 525.00 |
| **Magnesium (mg)** | 343.00 (252.50) | 280.00 (132.75) | **<0.001** | 270.00 | 200.00 |
| **Iron (mg)** | 14.40 (10.40) | 12.45 (5.95) | **<0.001** | 14.80 | 11.40 |
| **Zinc (mg)** | 12.00 (8.10) | 10.00 (4.78) | **<0.001** | 7.00 | 5.50 |
| **Selenium (µg)** | 154.00 (90.50) | 137.00 (73.25) | **<0.001** | 60.00 |  |
| **Iodine (µg)** | 232.00 (226.50) | 181.50 (134.75) | **<0.001** | 140.00 |  |
| **Vitamin A (µg)** | 753.00 (700.00) | 609.50 (502.25) | **<0.001** | 700.00 |  |
| **Vitamin B6 (mg)** | 2.70 (1.93) | 2.25 (1.20) | **<0.001** |  | 1.20 |
| **Vitamin B12 (µg)** | 9.30 (7.30) | 8.20 (4.88) | **<0.001** |  | 1.20 |
| **Vitamin C (mg)** | 231.00 (250.50) | 186.50 (161.25) | **<0.001** | 50.00 |  |
| **Vitamin D (µg)** | 5.30 (4.75) | 4.70 (3.10) | **<0.001** | 10.00 |  |
| **Thiamin (mg)** | 1.90 (1.30) | 1.55 (0.70) | **<0.001** | 0.90 |  |
| **Riboflavin (mg)** | 2.50 (2.25) | 2.00 (1.28) | **<0.001** | 1.40 |  |
| **Niacin (mg)** | 22.90 (15.95) | 19.80 (11.35) | **<0.001** | 12.00 |  |
| **Folate (µg)** | 354.00 (275.50) | 292.00 (159.25) | **<0.001** | 300.00 |  |
| *p-value for differences between all participants and plausible reporters only determined by Mann Whitney U test; p<0.05 considered statistically significant;* *DRV: Dietary Reference Value; RNI: Reference Nutrient Intake, EAR: Estimated Average Requirement; CHO: Carbohydrates.* | | | | | |
|  |  |  |  |  |  |
