# Supplementary material for: Dietary Determinants of Polyunsaturated Fatty Acid (PUFA) Status in a High Fish-Eating Cohort during Pregnancy
Source: Nutrients. 2018 Jul 20;10(7):927. doi: 10.3390/nu10070927 (PMC6073891; doi:10.3390/nu10070927)
Supplement: Supplementary file 1 [file nutrients-10-00927-s001.zip › SCDS NC2 FINAL FFQ.pdf]

SEYCHELLES CHILD DEVELOPMENT STUDY NC2
DIET QUESTIONNAIRE

Surname .... Subject ID ……………….. 1-8

First Name(s) ....

Address .... Questionnaire No ………. 9

.... Group Code …………….. 10

.... Survey …………………... 11-12

Phone …………………………………………... Female 13

Date of Birth .... Date of Survey 14-15

16-17

18-21

22-23

24-25

26-29

The following questions are about the foods you usually eat. Please indicate how often you had each food/drink, on average, DURING THE PAST 6 MONTHS. Circle the answer as in this example:

9 Never
8 1 time per month or less
7 2-3 times per month
6 1 time per week
5 2-4 times per week (circled)
4 5-6 times per week
3 1 time per day
2 2-3 times per day
1 4-5 times per day

If your answer is 2-4 times per week, circle '5', as shown.

PLEASE ANSWER EVERY QUESTION

BEVERAGES

Please enter your AVERAGE total use, DURING THE PAST 6 MONTHS, of each specified beverage

1. Pure fruit juice bought from shop e.g. Liquifruit, Ceres or homemade juice e.g.lime, orange, mango..

9 Never
8 1 time per month or less
7 2-3 times per month
6 1 time per week
5 2-4 times per week
4 5-6 times per week
3 1 time per day
2 2-3 times per day
1 4-5 times per day

30

Each time you drink pure fruit juice, how much do you usually drink? 31

1. Less than one glass
2. One glass
3. One carton (250ml)
4. One can (330 ml)

2. Water (tap water, bottled water)

9 Never
8 1 time per month or less
7 2-3 times per month
6 1 time per week
5 2-4 times per week
4 5-6 times per week
3 1 time per day
2 2-3 times per day
1 4-5 times per day

32

Each time you drink water, how much do you usually drink? 33

1. Less than one glass
2. One glass
3. One bottle

3. Fizzy drinks e.g. cola, lemonade, soda, sprite (diet or non-diet)

**9** Never	**4** 5-6 times per week
**8** 1 time per month or less	**3** 1 time per day
**7** 2-3 times per month	**2** 2-3 times per day
**6** 1 time per week	**1** 4-5 times per day
**5** 2-4 times per week	

☐ 34

Each time you have a fizzy drink, how much do you usually drink?

1. Less than one glass
2. One glass
3. One small bottle (300ml)
4. One can (330ml)
5. One small pet bottle (500ml)

☐ 35

4. Tea/Coffee

**9** Never	**4** 5-6 times per week
**8** 1 time per month or less	**3** 1 time per day
**7** 2-3 times per month	**2** 2-3 times per day
**6** 1 time per week	**1** 4-5 times per day
**5** 2-4 times per week	

☐ 36

When you drink tea/coffee, how much do you have at a time?

1. One cup
2. One mug
3. Two or more cups
4. Two or more mugs

☐ 37

5. What milk would you usually add to your tea/coffee (please choose one)?

1. None
2. Semi-skimmed liquid milk e.g. Slimilk (SMB), Rainbow
3. Full cream liquid milk
4. Skimmed powdered (Anlene)
5. Semi-skimmed powdered (Anmum)
6. Full cream powdered (Nido, Melody, Anchor, Coast)
7. Evaporated milk
8. Condensed milk
9. Skimmed (non-fat) liquid milk

☐ 38

6. How much sugar would you usually add to your tea/coffee? .... teaspoons

☐ 39

7. Drinking chocolate/ovaltine/milo/horlicks/bournvita/nesquik (cup)

**9** Never	**4** 5-6 times per week
**8** 1 time per month or less	**3** 1 time per day
**7** 2-3 times per month	**2** 2-3 times per day
**6** 1 time per week	**1** 4-5 times per day
**5** 2-4 times per week	

☐ 40

8. What milk would you usually add to your chocolate (please choose one)?

1. None
2. Semi-skimmed liquid milk e.g. Slimilk (SMB), Rainbow
3. Full cream liquid milk
4. Skimmed powdered (Anlene)
5. Semi-skimmed powdered (Anmum)
6. Full cream powdered (Nido, Melody, Anchor, Coast)
7. Evaporated milk
8. Condensed milk
9. Skimmed (non-fat) liquid milk

☐ 41

9. How much sugar would you usually add to your chocolate?................. teaspoons

☐ 42

10. Milk

9 Never
8 1 time per month or less
7 2-3 times per month
6 1 time per week
5 2-4 times per week
4 5-6 times per week
3 1 time per day
2 2-3 times per day
1 4-5 times per day

43

Each time you drink milk, how much do you usually drink (glasses)?.............................. 44

What type of milk would you usually drink? 45

1. Semi skimmed milk e.g. Slimilk (SMB)
2. Full cream liquid milk
3. Skimmed powdered, made up with water e.g. Anlene
4. Semi skimmed powdered, made up with water
5. Full cream powdered made up with water e.g. Nido, Melody, An
6. Flavoured milk e.g. perette, milo
7. Other (please specify) ……………………………………………..

11. Alcohol
(bottle/can/glass)

9 Never
8 1 time per month or less
7 2-3 times per month
6 1 time per week
5 2-4 times per week
4 5-6 times per week
3 1 time per day
2 2-3 times per day
1 4-5 times per day

46

Each time you drink alcohol, how much do you usually drink? 47

1. Less than one glass
2. One glass
3. One bottle/can
4. Two bottles/cans
5. Three bottles/cans
6. Four bottles/cans
7. Five or more bottles/cans

State the type(s) of alcohol that you USUALLY consume? 48

FRUITS

Please enter your AVERAGE total use, DURING THE PAST 6 MONTHS, of each specified fruit

12. Mangoes

9 Never
8 1 time per month or less
7 2-3 times per month
6 1 time per week
5 2-4 times per week
4 5-6 times per week
3 1 time per day
2 2-3 times per day
1 4-5 times per day

49

13. Bananas

9 Never
8 1 time per month or less
7 2-3 times per month
6 1 time per week
5 2-4 times per week
4 5-6 times per week
3 1 time per day
2 2-3 times per day
1 4-5 times per day

50

14. Apples

9 Never
8 1 time per month or less
7 2-3 times per month
6 1 time per week
5 2-4 times per week
4 5-6 times per week
3 1 time per day
2 2-3 times per day
1 4-5 times per day

51

15. Custard apple/sweetsop/ox-heart
(zat, serimolya, ker-d-bef)

9 Never
8 1 time per month or less
7 2-3 times per month
6 1 time per week
5 2-4 times per week
4 5-6 times per week
3 1 time per day
2 2-3 times per day
1 4-5 times per day

52

16. Oranges/mandarin/grapefruit/citron

**9**	Never	**4**	5-6 times per week	☐ 53
**8**	1 time per month or less	**3**	1 time per day	
**7**	2-3 times per month	**2**	2-3 times per day	
**6**	1 time per week	**1**	4-5 times per day	
**5**	2-4 times per week			

17. Guava

**9**	Never	**4**	5-6 times per week	☐ 54
**8**	1 time per month or less	**3**	1 time per day	
**7**	2-3 times per month	**2**	2-3 times per day	
**6**	1 time per week	**1**	4-5 times per day	
**5**	2-4 times per week			

18. Papaya

**9**	Never	**4**	5-6 times per week	☐ 55
**8**	1 time per month or less	**3**	1 time per day	
**7**	2-3 times per month	**2**	2-3 times per day	
**6**	1 time per week	**1**	4-5 times per day	
**5**	2-4 times per week			

19. Jamalac

**9**	Never	**4**	5-6 times per week	☐ 56
**8**	1 time per month or less	**3**	1 time per day	
**7**	2-3 times per month	**2**	2-3 times per day	
**6**	1 time per week	**1**	4-5 times per day	
**5**	2-4 times per week			

20. Passion fruit

**9**	Never	**4**	5-6 times per week	☐ 57
**8**	1 time per month or less	**3**	1 time per day	
**7**	2-3 times per month	**2**	2-3 times per day	
**6**	1 time per week	**1**	4-5 times per day	
**5**	2-4 times per week			

21. Star fruit
(karanbol)

**9**	Never	**4**	5-6 times per week	☐ 58
**8**	1 time per month or less	**3**	1 time per day	
**7**	2-3 times per month	**2**	2-3 times per day	
**6**	1 time per week	**1**	4-5 times per day	
**5**	2-4 times per week			

22. Melon

**9**	Never	**4**	5-6 times per week	☐ 59
**8**	1 time per month or less	**3**	1 time per day	
**7**	2-3 times per month	**2**	2-3 times per day	
**6**	1 time per week	**1**	4-5 times per day	
**5**	2-4 times per week			

23. Jackfruit (zak)

**9**	Never	**4**	5-6 times per week	☐ 60
**8**	1 time per month or less	**3**	1 time per day	
**7**	2-3 times per month	**2**	2-3 times per day	
**6**	1 time per week	**1**	4-5 times per day	
**5**	2-4 times per week			

24. Canned fruit cocktail
(fri-o-zi dan bwat)

**9**	Never	**4**	5-6 times per week	☐ 61
**8**	1 time per month or less	**3**	1 time per day	
**7**	2-3 times per month	**2**	2-3 times per day	
**6**	1 time per week	**1**	4-5 times per day	
**5**	2-4 times per week			

25. Dried fruits, e.g. sultanas,
raisins, dates

**9**	Never	**4**	5-6 times per week	☐ 62
**8**	1 time per month or less	**3**	1 time per day	
**7**	2-3 times per month	**2**	2-3 times per day	
**6**	1 time per week	**1**	4-5 times per day	
**5**	2-4 times per week			

26. Other fruit e.g.kiwi, apricots, peaches

**9** Never	**4** 5-6 times per week	☐ 63
**8** 1 time per month or less	**3** 1 time per day	
**7** 2-3 times per month	**2** 2-3 times per day	
**6** 1 time per week	**1** 4-5 times per day	
**5** 2-4 times per week		

27. In summary, how often do you eat fruit?

**9** Never	**4** 5-6 times per week	☐ 64
**8** 1 time per month or less	**3** 1 time per day	
**7** 2-3 times per month	**2** 2-3 times per day	
**6** 1 time per week	**1** 4-5 times per day	
**5** 2-4 times per week		

VEGETABLES

Please enter your AVERAGE total use, DURING THE PAST 6 MONTHS, of each specified vegetable

28. Bilimbi (bilenbi)

**9** Never	**4** 5-6 times per week	☐ 65
**8** 1 time per month or less	**3** 1 time per day	
**7** 2-3 times per month	**2** 2-3 times per day	
**6** 1 time per week	**1** 4-5 times per day	
**5** 2-4 times per week		

29. Margoz, patol, kalbas, pousinika

**9** Never	**4** 5-6 times per week	☐ 66
**8** 1 time per month or less	**3** 1 time per day	
**7** 2-3 times per month	**2** 2-3 times per day	
**6** 1 time per week	**1** 4-5 times per day	
**5** 2-4 times per week		

30. Cabbage (sou)

**9** Never	**4** 5-6 times per week	☐ 67
**8** 1 time per month or less	**3** 1 time per day	
**7** 2-3 times per month	**2** 2-3 times per day	
**6** 1 time per week	**1** 4-5 times per day	
**5** 2-4 times per week		

31. Carrots

**9** Never	**4** 5-6 times per week	☐ 68
**8** 1 time per month or less	**3** 1 time per day	
**7** 2-3 times per month	**2** 2-3 times per day	
**6** 1 time per week	**1** 4-5 times per day	
**5** 2-4 times per week		

32. Courgettes

**9** Never	**4** 5-6 times per week	☐ 69
**8** 1 time per month or less	**3** 1 time per day	
**7** 2-3 times per month	**2** 2-3 times per day	
**6** 1 time per week	**1** 4-5 times per day	
**5** 2-4 times per week		

33. Cucumber

**9** Never	**4** 5-6 times per week	☐ 70
**8** 1 time per month or less	**3** 1 time per day	
**7** 2-3 times per month	**2** 2-3 times per day	
**6** 1 time per week	**1** 4-5 times per day	
**5** 2-4 times per week		

34. Green beans (zariko)

**9** Never	**4** 5-6 times per week	☐ 71
**8** 1 time per month or less	**3** 1 time per day	
**7** 2-3 times per month	**2** 2-3 times per day	
**6** 1 time per week	**1** 4-5 times per day	
**5** 2-4 times per week		

35. Lentils, cooked

9 Never
8 1 time per month or less
7 2-3 times per month
6 1 time per week
5 2-4 times per week
4 5-6 times per week
3 1 time per day
2 2-3 times per day
1 4-5 times per day

❑ 72

36. Other pulses e.g. kidney beans, chick peas

9 Never
8 1 time per month or less
7 2-3 times per month
6 1 time per week
5 2-4 times per week
4 5-6 times per week
3 1 time per day
2 2-3 times per day
1 4-5 times per day

❑ 73

37. Lettuce

9 Never
8 1 time per month or less
7 2-3 times per month
6 1 time per week
5 2-4 times per week
4 5-6 times per week
3 1 time per day
2 2-3 times per day
1 4-5 times per day

❑ 74

38. Onions

9 Never
8 1 time per month or less
7 2-3 times per month
6 1 time per week
5 2-4 times per week
4 5-6 times per week
3 1 time per day
2 2-3 times per day
1 4-5 times per day

❑ 75

39. Peas (pti pwa)

9 Never
8 1 time per month or less
7 2-3 times per month
6 1 time per week
5 2-4 times per week
4 5-6 times per week
3 1 time per day
2 2-3 times per day
1 4-5 times per day

❑ 76

40. Capsicums (gro piman)

9 Never
8 1 time per month or less
7 2-3 times per month
6 1 time per week
5 2-4 times per week
4 5-6 times per week
3 1 time per day
2 2-3 times per day
1 4-5 times per day

❑ 77

41. Pumpkin

9 Never
8 1 time per month or less
7 2-3 times per month
6 1 time per week
5 2-4 times per week
4 5-6 times per week
3 1 time per day
2 2-3 times per day
1 4-5 times per day

❑ 78

42. Sousout

9 Never
8 1 time per month or less
7 2-3 times per month
6 1 time per week
5 2-4 times per week
4 5-6 times per week
3 1 time per day
2 2-3 times per day
1 4-5 times per day

❑ 79

43. Sweetcorn

9 Never
8 1 time per month or less
7 2-3 times per month
6 1 time per week
5 2-4 times per week
4 5-6 times per week
3 1 time per day
2 2-3 times per day
1 4-5 times per day

❑ 80

44. Tomatoes

9 Never
8 1 time per month or less
7 2-3 times per month
6 1 time per week
5 2-4 times per week
4 5-6 times per week
3 1 time per day
2 2-3 times per day
1 4-5 times per day

❑ 81

45. Tomato sauce/ketchup

9 Never
8 1 time per month or less
7 2-3 times per month
6 1 time per week
5 2-4 times per week
4 5-6 times per week
3 1 time per day
2 2-3 times per day
1 4-5 times per day

☐ 82

46. Chilli sauce, bottled

9 Never
8 1 time per month or less
7 2-3 times per month
6 1 time per week
5 2-4 times per week
4 5-6 times per week
3 1 time per day
2 2-3 times per day
1 4-5 times per day

☐ 83

47. Mixed vegetables

9 Never
8 1 time per month or less
7 2-3 times per month
6 1 time per week
5 2-4 times per week
4 5-6 times per week
3 1 time per day
2 2-3 times per day
1 4-5 times per day

☐ 84

48. Watercress (kreson)

9 Never
8 1 time per month or less
7 2-3 times per month
6 1 time per week
5 2-4 times per week
4 5-6 times per week
3 1 time per day
2 2-3 times per day
1 4-5 times per day

☐ 85

49. Other vegetables eg. mushrooms, beetroot, okra, broccoli…

9 Never
8 1 time per month or less
7 2-3 times per month
6 1 time per week
5 2-4 times per week
4 5-6 times per week
3 1 time per day
2 2-3 times per day
1 4-5 times per day

☐ 86

50. In summary, how often do you eat vegetables?

9 Never
8 1 time per month or less
7 2-3 times per month
6 1 time per week
5 2-4 times per week
4 5-6 times per week
3 1 time per day
2 2-3 times per day
1 4-5 times per day

☐ 87

FRICASSE/CHUTNEY DISHES

Please enter your AVERAGE total use, DURING THE PAST 6 MONTHS, of each specified dish

51. Fricasse (pumpkin, ‘kalbas, pousinika’, lantiy)

9 Never
8 1 time per month or less
7 2-3 times per month
6 1 time per week
5 2-4 times per week
4 5-6 times per week
3 1 time per day
2 2-3 times per day
1 4-5 times per day

☐ 88

52. Chutney (golden apple, mango, ‘sousout’, pawpaw, radish, eggplant,

9 Never
8 1 time per month or less
7 2-3 times per month
6 1 time per week
5 2-4 times per week
4 5-6 times per week
3 1 time per day
2 2-3 times per day
1 4-5 times per day

☐ 89

53. Fish chutney (includes salted fish chutney)

9 Never
8 1 time per month or less
7 2-3 times per month
6 1 time per week
5 2-4 times per week
4 5-6 times per week
3 1 time per day
2 2-3 times per day
1 4-5 times per day

☐ 90

POTATOES

Please enter your AVERAGE total use, DURING THE PAST 6 MONTHS, of each specified dish

54. Potato chips (home-made, Take-away...)

9 Never
8 1 time per month or less
7 2-3 times per month
6 1 time per week
5 2-4 times per week
4 5-6 times per week
3 1 time per day
2 2-3 times per day
1 4-5 times per day

❑ 91

55. Potato salad, boiled/roast potato, potato cooked in dishes

9 Never
8 1 time per month or less
7 2-3 times per month
6 1 time per week
5 2-4 times per week
4 5-6 times per week
3 1 time per day
2 2-3 times per day
1 4-5 times per day

❑ 92

56. Potato crisps e.g. Jacker, Pringles...

9 Never
8 1 time per month or less
7 2-3 times per month
6 1 time per week
5 2-4 times per week
4 5-6 times per week
3 1 time per day
2 2-3 times per day
1 4-5 times per day

❑ 93

SNACKS

Please enter your AVERAGE total use, DURING THE PAST 6 MONTHS, of each specified snack

57. Corn snacks e.g. twisties, NikNaks (packet)

9 Never
8 1 time per month or less
7 2-3 times per month
6 1 time per week
5 2-4 times per week
4 5-6 times per week
3 1 time per day
2 2-3 times per day
1 4-5 times per day

❑ 94

58. Nuts e.g peanuts, cashews, macademia nuts

9 Never
8 1 time per month or less
7 2-3 times per month
6 1 time per week
5 2-4 times per week
4 5-6 times per week
3 1 time per day
2 2-3 times per day
1 4-5 times per day

❑ 95

59. Chilli cakes (one)

9 Never
8 1 time per month or less
7 2-3 times per month
6 1 time per week
5 2-4 times per week
4 5-6 times per week
3 1 time per day
2 2-3 times per day
1 4-5 times per day

❑ 96

60. Meat patties (one) (pate/boulet pwason)

9 Never
8 1 time per month or less
7 2-3 times per month
6 1 time per week
5 2-4 times per week
4 5-6 times per week
3 1 time per day
2 2-3 times per day
1 4-5 times per day

❑ 97

61. Local chips (cassava, breadfruit, banana (homemade/bought)

9 Never
8 1 time per month or less
7 2-3 times per month
6 1 time per week
5 2-4 times per week
4 5-6 times per week
3 1 time per day
2 2-3 times per day
1 4-5 times per day

❑ 98

62. Moutay

9 Never
8 1 time per month or less
7 2-3 times per month
6 1 time per week
5 2-4 times per week
4 5-6 times per week
3 1 time per day
2 2-3 times per day
1 4-5 times per day

❑ 99

63. Moulouk

9 Never
8 1 time per month or less
7 2-3 times per month
6 1 time per week
5 2-4 times per week
4 5-6 times per week
3 1 time per day
2 2-3 times per day
1 4-5 times per day

❑ 100

64. Samosa

9 Never
8 1 time per month or less
7 2-3 times per month
6 1 time per week
5 2-4 times per week
4 5-6 times per week
3 1 time per day
2 2-3 times per day
1 4-5 times per day

❑ 101

What would usually be in the samosa (please select one)?

1. Meat
2. Fish
3. Vegetables only

❑ 102

DAIRY

Please enter your AVERAGE total use, DURING THE PAST 6 MONTHS, of each specified item

65. Custard

9 Never
8 1 time per month or less
7 2-3 times per month
6 1 time per week
5 2-4 times per week
4 5-6 times per week
3 1 time per day
2 2-3 times per day
1 4-5 times per day

❑ 103

66. What milk would you usually use to make custard?

1. None
2. Semi-skimmed liquid milk e.g. Slimilk (SMB), Rainbow
3. Full cream liquid milk
4. Skimmed powdered (Anlene)
5. Semi-skimmed powdered (Anmum)
6. Full cream powdered (Nido, Melody, Anchor, Coast)
7. Evaporated milk
8. Condensed milk
9. Skimmed (non-fat) liquid milk

❑ 104

67. Ice cream

9 Never
8 1 time per month or less
7 2-3 times per month
6 1 time per week
5 2-4 times per week
4 5-6 times per week
3 1 time per day
2 2-3 times per day
1 4-5 times per day

❑ 105

68. Yogurt

9 Never
8 1 time per month or less
7 2-3 times per month
6 1 time per week
5 2-4 times per week
4 5-6 times per week
3 1 time per day
2 2-3 times per day
1 4-5 times per day

❑ 106

69. Cheese

9 Never
8 1 time per month or less
7 2-3 times per month
6 1 time per week
5 2-4 times per week
4 5-6 times per week
3 1 time per day
2 2-3 times per day
1 4-5 times per day

❑ 107

70. Mayonnaise

9 Never
8 1 time per month or less
7 2-3 times per month
6 1 time per week
5 2-4 times per week
4 5-6 times per week
3 1 time per day
2 2-3 times per day
1 4-5 times per day

❑ 108

EGGS and MEAT

Please enter your AVERAGE total use, DURING THE PAST 6 MONTHS, of each specified item

71. Eggs (one whole)

9 Never
8 1 time per month or less
7 2-3 times per month
6 1 time per week
5 2-4 times per week
4 5-6 times per week
3 1 time per day
2 2-3 times per day
1 4-5 times per day

☐ 109

72. Bacon (two slices)

9 Never
8 1 time per month or less
7 2-3 times per month
6 1 time per week
5 2-4 times per week
4 5-6 times per week
3 1 time per day
2 2-3 times per day
1 4-5 times per day

☐ 110

73. Sausages - meat (one)

9 Never
8 1 time per month or less
7 2-3 times per month
6 1 time per week
5 2-4 times per week
4 5-6 times per week
3 1 time per day
2 2-3 times per day
1 4-5 times per day

☐ 111

74. Creole sausages or frankfurters (one)

9 Never
8 1 time per month or less
7 2-3 times per month
6 1 time per week
5 2-4 times per week
4 5-6 times per week
3 1 time per day
2 2-3 times per day
1 4-5 times per day

☐ 112

75. Burgers – meat

9 Never
8 1 time per month or less
7 2-3 times per month
6 1 time per week
5 2-4 times per week
4 5-6 times per week
3 1 time per day
2 2-3 times per day
1 4-5 times per day

☐ 113

76. Presack/ham/salami (one slice)

9 Never
8 1 time per month or less
7 2-3 times per month
6 1 time per week
5 2-4 times per week
4 5-6 times per week
3 1 time per day
2 2-3 times per day
1 4-5 times per day

☐ 114

77. Pate (spread on bread/rolls/ biscuits)
`

9 Never
8 1 time per month or less
7 2-3 times per month
6 1 time per week
5 2-4 times per week
4 5-6 times per week
3 1 time per day
2 2-3 times per day
1 4-5 times per day

☐ 115

78. Processed meat, e.g. corned beef/luncheon meat

9 Never
8 1 time per month or less
7 2-3 times per month
6 1 time per week
5 2-4 times per week
4 5-6 times per week
3 1 time per day
2 2-3 times per day
1 4-5 times per day

☐ 116

79. Chicken

9 Never
8 1 time per month or less
7 2-3 times per month
6 1 time per week
5 2-4 times per week
4 5-6 times per week
3 1 time per day
2 2-3 times per day
1 4-5 times per day

☐ 117

80. If you eat chicken, do you eat the skin? Yes / No

☐ 118

81. Beef

9 Never
8 1 time per month or less
7 2-3 times per month
6 1 time per week
5 2-4 times per week
4 5-6 times per week
3 1 time per day
2 2-3 times per day
1 4-5 times per day

119

82. Pork

9 Never
8 1 time per month or less
7 2-3 times per month
6 1 time per week
5 2-4 times per week
4 5-6 times per week
3 1 time per day
2 2-3 times per day
1 4-5 times per day

120

83. Lamb

9 Never
8 1 time per month or less
7 2-3 times per month
6 1 time per week
5 2-4 times per week
4 5-6 times per week
3 1 time per day
2 2-3 times per day
1 4-5 times per day

121

84. Pasta (macaroni, spaghetti, lasagne) cooked with meat/meat products e.g.minced meat, corned beef, ham, bacon...

9 Never
8 1 time per month or less
7 2-3 times per month
6 1 time per week
5 2-4 times per week
4 5-6 times per week
3 1 time per day
2 2-3 times per day
1 4-5 times per day

122

85. Noodles cooked with meat/meat products

9 Never
8 1 time per month or less
7 2-3 times per month
6 1 time per week
5 2-4 times per week
4 5-6 times per week
3 1 time per day
2 2-3 times per day
1 4-5 times per day

123

86. Meat organs (eg. liver, heart, kidneys, giblets)

9 Never
8 1 time per month or less
7 2-3 times per month
6 1 time per week
5 2-4 times per week
4 5-6 times per week
3 1 time per day
2 2-3 times per day
1 4-5 times per day

124

87. Black pudding

9 Never
8 1 time per month or less
7 2-3 times per month
6 1 time per week
5 2-4 times per week
4 5-6 times per week
3 1 time per day
2 2-3 times per day
1 4-5 times per day

125

CEREALS and BREADS

Please enter your AVERAGE total use, DURING THE PAST 6 MONTHS, of each specified item

88. Oatmeal/porridge (bowl)

9 Never
8 1 time per month or less
7 2-3 times per month
6 1 time per week
5 2-4 times per week
4 5-6 times per week
3 1 time per day
2 2-3 times per day
1 4-5 times per day

126

How much would you usually eat?

1. Small
2. Medium
3. Large

127

89. What do you usually add to your oatmeal (please choose one)? ☐ 128

1. None
2. Semi-skimmed liquid milk e.g. Slimilk (SMB), Rainbow
3. Full cream liquid milk
4. Skimmed powdered (Anlene)
5. Semi-skimmed powdered (Anmum)
6. Full cream powdered (Nido, Melody, Anchor, Coast)
7. Evaporated milk
8. Condensed milk
9. Skimmed (non-fat) liquid milk

90. Other cereal e.g. cornflakes, weetabix, muesli, rice krispies, cerelac ☐ 129

9 Never
8 1 time per month or less
7 2-3 times per month
6 1 time per week
5 2-4 times per week
4 5-6 times per week
3 1 time per day
2 2-3 times per day
1 4-5 times per day

91. Which other type of cereal would you have? .... ☐ 130

92. What type of milk would you usually add (please choose one)? ☐ 131

1. None
2. Semi-skimmed liquid milk e.g. Slimilk (SMB), Rainbow
3. Full cream liquid milk
4. Skimmed powdered (Anlene)
5. Semi-skimmed powdered (Anmum)
6. Full cream powdered (Nido, Melody, Anchor, Coast)
7. Evaporated milk
8. Condensed milk
9. Skimmed (non-fat) liquid milk

93. White bread (slice, roll, baguette) ☐ 132

9 Never
8 1 time per month or less
7 2-3 times per month
6 1 time per week
5 2-4 times per week
4 5-6 times per week
3 1 time per day
2 2-3 times per day
1 4-5 times per day

94. Brown/granary bread ☐ 133

9 Never
8 1 time per month or less
7 2-3 times per month
6 1 time per week
5 2-4 times per week
4 5-6 times per week
3 1 time per day
2 2-3 times per day
1 4-5 times per day

95. Plain rice ☐ 134

9 Never
8 1 time per month or less
7 2-3 times per month
6 1 time per week
5 2-4 times per week
4 5-6 times per week
3 1 time per day
2 2-3 times per day
1 4-5 times per day

How many spoons would you have? ☐ 135

1. 2-3 tablespoons
2. 4-5 tablespoons
3. 6-8 tablespoons
4. more than 8 tablespoons
5. 1 rice cooker spoon
6. 2 rice cooker spoons
7. 3 rice cooker spoons
8. 4 or more rice cooker spoons

96. Fried rice ☐ 136

9 Never
8 1 time per month or less
7 2-3 times per month
6 1 time per week
5 2-4 times per week
4 5-6 times per week
3 1 time per day
2 2-3 times per day
1 4-5 times per day

How many spoons of fried rice would you have? 137

1. 2-3 tablespoons
2. 4-5 tablespoons
3. 6-8 tablespoons
4. more than 8 tablespoons
5. 1 rice cooker spoon
6. 2 rice cooker spoons
7. 3 rice cooker spoons
8. 4 or more rice cooker spoons

97. Pizza 138

9 Never
8 1 time per month or less
7 2-3 times per month
6 1 time per week
5 2-4 times per week
4 5-6 times per week
3 1 time per day
2 2-3 times per day
1 4-5 times per day

98. When you have pizza, how many slices would you usually eat? 139

1. One
2. Two
3. Three
4. Four
5. Five
6. Six or more

BOUILLON DISHES

Please enter your AVERAGE total use, DURING THE PAST 6 MONTHS, of each specified dish

99. Bouillon bred with fish 140

9 Never
8 1 time per month or less
7 2-3 times per month
6 1 time per week
5 2-4 times per week
4 5-6 times per week
3 1 time per day
2 2-3 times per day
1 4-5 times per day

100. For your bouillon bred, which fish do you mostly have and which oil do you generally use? (please select one option only) 141

1. Shoemaker (kordonnyen), use palm oil e.g. turkey
2. Red snapper (bourzwa), use palm oil e.g turkey
3. Grouper (vyey), use palm oil e.g turkey
4. Other fish, use palm oil e.g turkey
5. Shoemaker (kordonnyen), use other oil e.g sunflower, olive
6. Red snapper (bourzwa), use other oil e.g. sunflower, olive
7. Grouper (vyey), use other oil e.g. sunflower, olive
8. Other fish, use other oil e.g sunflower, olive

101. Bouillon bred (no fish) 142

9 Never
8 1 time per month or less
7 2-3 times per month
6 1 time per week
5 2-4 times per week
4 5-6 times per week
3 1 time per day
2 2-3 times per day
1 4-5 times per day

102. When you have bouillon bred (no fish), which oil do you generally use? 143

1. Palm oil e.g. turkey oil
2. Other oil e.g sunflower/olive
3. No oil

103. Bouillon blan 144

9 Never
8 1 time per month or less
7 2-3 times per month
6 1 time per week
5 2-4 times per week
4 5-6 times per week
3 1 time per day
2 2-3 times per day
1 4-5 times per day

104. For your bouillon blan, which fish do you mostly have and which oil do you generally use? (please select one option only) 145

1. shoemaker (kordonnyen), palm oil e.g turkey
2. red snapper (bourzwa), palm oil e.g turkey
3. grouper (vyey), palm oil e.g turkey oil
4. other fish, palm oil e.g. turkey oil
5. shoemaker (kordonnyen), other oil e.g sunflower, olive
6. red snapper (bourzwa), other oil e.g. sunflower, olive
7. grouper (vyey), other oil e.g. sunflower, olive
8. other fish, other oil e.g. sunflower, olive

FISH

Please enter your AVERAGE total use, DURING THE PAST 6 MONTHS, of each specified fish (one fillet fried/grilled)

105. Karang — 146

9 Never
8 1 time per month or less
7 2-3 times per month
6 1 time per week
5 2-4 times per week
4 5-6 times per week
3 1 time per day
2 2-3 times per day
1 4-5 times per day

106. Barracuda (Bekin, Tazar) — 147

9 Never
8 1 time per month or less
7 2-3 times per month
6 1 time per week
5 2-4 times per week
4 5-6 times per week
3 1 time per day
2 2-3 times per day
1 4-5 times per day

107. Tuna (Ton) fresh, not canned — 148

9 Never
8 1 time per month or less
7 2-3 times per month
6 1 time per week
5 2-4 times per week
4 5-6 times per week
3 1 time per day
2 2-3 times per day
1 4-5 times per day

108. Red snapper (Bourzwa, Etelis, Varavara, Terez — 149

9 Never
8 1 time per month or less
7 2-3 times per month
6 1 time per week
5 2-4 times per week
4 5-6 times per week
3 1 time per day
2 2-3 times per day
1 4-5 times per day

109. Grouper (Vyey) — 150

9 Never
8 1 time per month or less
7 2-3 times per month
6 1 time per week
5 2-4 times per week
4 5-6 times per week
3 1 time per day
2 2-3 times per day
1 4-5 times per day

110. Parrot fish (Kakatwa, Marar) — 151

9 Never
8 1 time per month or less
7 2-3 times per month
6 1 time per week
5 2-4 times per week
4 5-6 times per week
3 1 time per day
2 2-3 times per day
1 4-5 times per day

111. Spinefoot shoemaker (Kordonyen) — 152

9 Never
8 1 time per month or less
7 2-3 times per month
6 1 time per week
5 2-4 times per week
4 5-6 times per week
3 1 time per day
2 2-3 times per day
1 4-5 times per day

112. Mackerel (Makro)

9 Never
8 1 time per month or less
7 2-3 times per month
6 1 time per week
5 2-4 times per week
4 5-6 times per week
3 1 time per day
2 2-3 times per day
1 4-5 times per day

☐ 153

113. Emperor fish (Baksou, Madanm Beri, Kaptenn, Gel long, Zekler, Ziblo)

9 Never
8 1 time per month or less
7 2-3 times per month
6 1 time per week
5 2-4 times per week
4 5-6 times per week
3 1 time per day
2 2-3 times per day
1 4-5 times per day

☐ 154

114. Job (Zob)

9 Never
8 1 time per month or less
7 2-3 times per month
6 1 time per week
5 2-4 times per week
4 5-6 times per week
3 1 time per day
2 2-3 times per day
1 4-5 times per day

☐ 155

115. Bonito (Bonit)

9 Never
8 1 time per month or less
7 2-3 times per month
6 1 time per week
5 2-4 times per week
4 5-6 times per week
3 1 time per day
2 2-3 times per day
1 4-5 times per day

☐ 156

116. Octopus

9 Never
8 1 time per month or less
7 2-3 times per month
6 1 time per week
5 2-4 times per week
4 5-6 times per week
3 1 time per day
2 2-3 times per day
1 4-5 times per day

☐ 157

117. Tinned tuna

9 Never
8 1 time per month or less
7 2-3 times per month
6 1 time per week
5 2-4 times per week
4 5-6 times per week
3 1 time per day
2 2-3 times per day
1 4-5 times per day

☐ 158

118. Tinned sardines

9 Never
8 1 time per month or less
7 2-3 times per month
6 1 time per week
5 2-4 times per week
4 5-6 times per week
3 1 time per day
2 2-3 times per day
1 4-5 times per day

☐ 159

119. Shark (reken)

9 Never
8 1 time per month or less
7 2-3 times per month
6 1 time per week
5 2-4 times per week
4 5-6 times per week
3 1 time per day
2 2-3 times per day
1 4-5 times per day

☐ 160

120. Salted fish

9 Never
8 1 time per month or less
7 2-3 times per month
6 1 time per week
5 2-4 times per week
4 5-6 times per week
3 1 time per day
2 2-3 times per day
1 4-5 times per day

☐ 161

121. Prawns

9 Never
8 1 time per month or less
7 2-3 times per month
6 1 time per week
5 2-4 times per week
4 5-6 times per week
3 1 time per day
2 2-3 times per day
1 4-5 times per day

☐ 162

122. Crab/lobster

9 Never
8 1 time per month or less
7 2-3 times per month
6 1 time per week
5 2-4 times per week
4 5-6 times per week
3 1 time per day
2 2-3 times per day
1 4-5 times per day

163

123. Other fish, e.g.swordfish sirizyen, madanm beri,sardin, rouze, dorad,galate,marlin kingfish, etc..

9 Never
8 1 time per month or less
7 2-3 times per month
6 1 time per week
5 2-4 times per week
4 5-6 times per week
3 1 time per day
2 2-3 times per day
1 4-5 times per day

164

SWEETS, CAKES, DESSERTS and OTHER FOODS

Please enter your AVERAGE total use, DURING THE PAST 6 MONTHS, of each specified food

124. Chocolate (bar or packet) e.g. dairy milk, wonder bar

9 Never
8 1 time per month or less
7 2-3 times per month
6 1 time per week
5 2-4 times per week
4 5-6 times per week
3 1 time per day
2 2-3 times per day
1 4-5 times per day

165

125. Sweets (packet) e.g. boiled sweets, pastilles

9 Never
8 1 time per month or less
7 2-3 times per month
6 1 time per week
5 2-4 times per week
4 5-6 times per week
3 1 time per day
2 2-3 times per day
1 4-5 times per day

166

126. Biscuits
,

9 Never
8 1 time per month or less
7 2-3 times per month
6 1 time per week
5 2-4 times per week
4 5-6 times per week
3 1 time per day
2 2-3 times per day
1 4-5 times per day

167

127. When you have biscuits, how many would you have each time?

1. one - two
2. three - four
3. five - six
4. seven - eight
5. nine or more

168

128. Feyte/tourt/danish

9 Never
8 1 time per month or less
7 2-3 times per month
6 1 time per week
5 2-4 times per week
4 5-6 times per week
3 1 time per day
2 2-3 times per day
1 4-5 times per day

169

129. Fruit cake/sponge cake/vanilla slice/other cake

9 Never
8 1 time per month or less
7 2-3 times per month
6 1 time per week
5 2-4 times per week
4 5-6 times per week
3 1 time per day
2 2-3 times per day
1 4-5 times per day

170

130. Trifle

9 Never
8 1 time per month or less
7 2-3 times per month
6 1 time per week
5 2-4 times per week
4 5-6 times per week
3 1 time per day
2 2-3 times per day
1 4-5 times per day

171

131. Ladob

**9** Never	**4** 5-6 times per week
**8** 1 time per month or less	**3** 1 time per day
**7** 2-3 times per month	**2** 2-3 times per day
**6** 1 time per week	**1** 4-5 times per day
**5** 2-4 times per week	

☐ 172

132. Creme brulee /crème caramel

**9** Never	**4** 5-6 times per week
**8** 1 time per month or less	**3** 1 time per day
**7** 2-3 times per month	**2** 2-3 times per day
**6** 1 time per week	**1** 4-5 times per day
**5** 2-4 times per week	

☐ 173

FATS AND OILS

Please enter your AVERAGE total use, DURING THE PAST 6 MONTHS, of each specified item

133. What do you usually spread on your bread (e.g. butter, margarine..)?

Type ….. Brand …... ☐ 174

134. How often do you have fried foods, e.g. chips, fish, bacon, sausages, eggs?

**9** Never	**4** 5-6 times per week
**8** 1 time per month or less	**3** 1 time per day
**7** 2-3 times per month	**2** 2-3 times per day
**6** 1 time per week	**1** 4-5 times per day
**5** 2-4 times per week	

☐ 175

135. What oil do you use for frying foods in? ☐ 176

1. Palm oil e.g. turkey oil
2. Other oil e.g sunflower/olive oil/soyabean oil

136. What oil do you use for cooking other than frying e.g curries/stews?

.... ☐ 177

137. What oil do you use for salads? .... ☐ 178

THANK YOU FOR COMPLETING THIS QUESTIONNAIRE
