# Supplementary material for: Dietary Determinants of Polyunsaturated Fatty Acid (PUFA) Status in a High Fish-Eating Cohort during Pregnancy
Source: Nutrients. 2018 Jul 20;10(7):927. doi: 10.3390/nu10070927 (PMC6073891; doi:10.3390/nu10070927)
Supplement: Supplementary file 1 [file nutrients-10-00927-s001.zip › SCDS NC2 FUQ.pdf]

SEYCHELLES CHILD DEVELOPMENT STUDY NC2
FISH USE QUESTIONNAIRE

Surname ……………………………………………… Subject ID …………………………………

First Name(s) ……………………………………… Date of birth …………………..…..……..

Address ………………………………………………. Phone ………………………….…..……….

…………………………………………………………. Date of survey ……………………………..

…………………………………………………………. Date of delivery if applicable ...……...……

Please tell us about the types of fish and fish products that you have eaten in **the last week**. Please fill in the table below. If you had fish in a curry or another mixed dish and you don't know its name, please just write "fish curry" in a blank space at the bottom of the "Name of fish" column.

**Name of fish**	**How many times did you eat it in the last week?**
Karang	
Barracuda (bekin)	
Fresh Tuna (ton)	
Red snapper (bourzwa)	
Grouper (vyey)	
Parrot fish (kakatwa)	
Spinefoot shoemaker (kordonnyen)	
Mackerel (makro)	
Emperor fish (baksou)	
Job (zob)	
Bonito (bonit)	
Octopus (zourit)	
Tinned tuna (ton dan bwat)	
Tinned sardines (sardine dan bwat)	
Shark (reken)	
Salted fish (pwason sale)	
Prawns (kanmaron)	
Crab/lobster (krab/oumar)	
Other fish - Please specify:	
Other fish - Please specify:	

If you are not currently pregnant, does this fish consumption reflect what you ate when you were pregnant?

Yes ☐
No ☐

If no, please say how much more or less fish you ate per week when you were pregnant.

……
……
……
