# Supplementary material for: Dietary Determinants of Polyunsaturated Fatty Acid (PUFA) Status in a High Fish-Eating Cohort during Pregnancy
Source: Nutrients. 2018 Jul 20;10(7):927. doi: 10.3390/nu10070927 (PMC6073891; doi:10.3390/nu10070927)
Supplement: Supplementary file 1 [file nutrients-10-00927-s001.zip › Supplementary Figure 1 - Scree plot .docx]

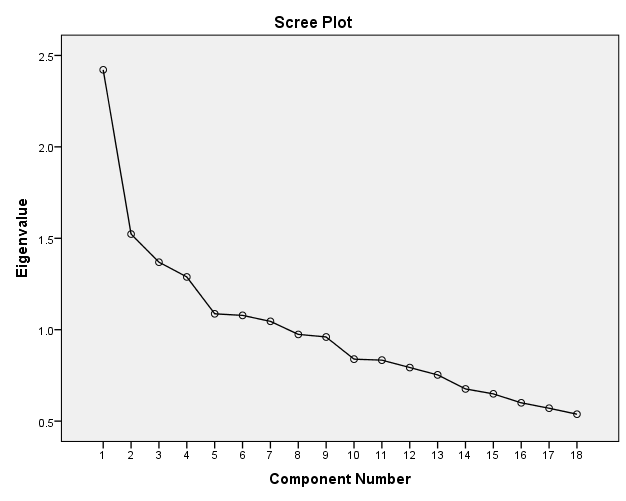


Supplementary Figure 1: Scree plot for identifying dietary patterns from principal component analysis
